# IMPlications of IMP2 in RNA Biology and Disease

**DOI:** 10.3390/ijms26062415

**Published:** 2025-03-07

**Authors:** Jessica Das, Ottavia Busia-Bourdain, Khizr M. Khan, Andrew L. Wolfe

**Affiliations:** 1 Department of Biological Sciences, Hunter College, City University of New York, New York, NY 10065, USA; 2 Molecular, Cellular, and Developmental Biology Subprogram of the Biology Ph.D. Program, Graduate Center, City University of New York, New York, NY 10031, USA; 3 New York Research and Mentoring for Postbaccalaureates (NY-RaMP) Program, Hunter College, New York, NY 10021, USA; 4 Biochemistry Ph.D. Program, Graduate Center, City University of New York, New York, NY 10031, USA; 5 Department of Pharmacology, Weill Cornell Medicine, New York, NY 10021, USA

**Keywords:** IMP2, *IGF2BP2*, RNA-binding protein, cancer, metabolism, immuno-oncology, m6A, RNA, modification, translation

## Abstract

Insulin-like growth factor 2 mRNA-binding protein 2 (IMP2) is an RNA-binding protein that positively regulates m6A-modified RNAs involved in critical cellular processes such as metabolism, oncogenesis, and immune function. Here, we elucidate facets of IMP2 biology, including several mechanisms of action on RNA, factors that regulate IMP2 expression, its relevant biological target RNAs, its role in normal development and disease, and its potential as a therapeutic target. IMP2 is a multi-level regulator of metabolism, influencing pathways linked to diabetes, obesity, and adipose function. Through genomic amplification and transcriptional overexpression in cancer cells, IMP2 can drive the initiation and progression of multiple cancer types, and high expression is associated with decreased overall survival of patients with cancer. IMP2 influences normal immune function, inflammation, macrophage polarization, and tumor immune evasion. IMP2 has emerged as a promising therapeutic target, particularly for cancers and metabolic diseases.

## 1. Introduction

**Figure 1:**
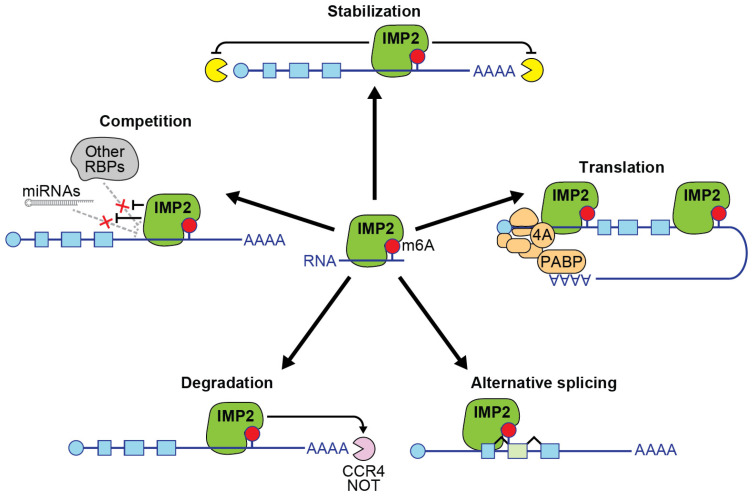
Mechanisms by which IMP2 regulates target genes. Promoting stabilization and translation are the most commonly reported mechanisms of IMP2 action, followed by competition, alternative splicing, and a report of mRNA degradation. The red circle represents methyl-6-A RNA modification, the blue line represents RNA, AAAA represents poly-A tail, the blue circles represent 7-methyl-G cap structure, the blue squares represent exons, green squares represent alternatively spliced exons, the yellow and pink circles with openings represent RNA degrading enzymes, PABP represents poly-A binding protein, the red Xs represent IMP2 presence preventing binding by other RBPs or miRNAs, which would otherwise bind and inhibit the target RNA. Figure created in Adobe Illustrator version 27.9.

RNA-binding proteins (RBPs) are a large and diverse group of proteins that help regulate essential processes encompassing mRNA transport, localization, alternative splicing, translation, and RNA stability [1]. RBPs play vital roles in cell biology by binding to messenger RNA and influencing their fate. This review focuses on a multi-functional RBP called insulin-like growth factor 2 mRNA-binding protein 2 (IMP2). The protein IMP2 is encoded by the gene *IGF2BP2*. IMP2 is well known for its role as an mRNA-binding protein that physically associates with its mRNA targets in an N^6^-methyladenosine (m6A)-dependent manner, leading to enhanced stability of target RNA and increased protein translation (Figure 1) [2]. IMP2 is known by several other names: VICKZ2, P62, and KH domain-containing protein overexpressed in cancer protein (KOC) [2,3]. It was identified in 1999 by the Nielson group, who used UV crosslinking and mobility shift assays to discover RNA-binding proteins involved in the translation of the *IGF-II leader 3* mRNA [4]. Shortly thereafter, the Zhang group isolated an *IGF2BP2* cDNA encoding a protein with a predicted mass of 66 kDa, which was nearly identical to the p62 protein but included an additional insertion of 43 amino acids, representing a second splice isoform of *IGF2BP2* [5].

In this review, we explore recent studies about key facets of IMP2 biology, including how IMP2 differs from IMP1 and IMP3, its mechanisms of action, regulation of its expression, characterized targets, and disease relevance across various biological contexts. IMP2 is known to function as a multi-level regulator of metabolism in adipose tissue, obesity, and diabetes. In cancer, IMP2 serves as a pivotal player in multiple cancer types, including colorectal adenocarcinomas, breast adenocarcinomas, leukemia, lymphoma, hepatocellular carcinomas, lung adenocarcinomas, ovarian carcinomas, endometrial cancer, oral cancer, renal cell carcinoma, thyroid cancers, and glioblastoma. By regulating m6A-modified RNAs associated with oncogenic function and metabolism, IMP2 drives disease progression, offering a promising opportunity for therapeutic targeting in both select cancers and metabolic disorders. Further, IMP2-regulated RNAs present a clear target for immuno-oncology, while pharmacological inhibition of IMP2 is emerging as a strategy to mitigate the myriad effects of this versatile RNA-binding protein—particularly as a regulator of type 2 diabetes and as a cancer-promoting oncoprotein that governs a broad spectrum of oncogenic RNAs.

### 1.1. IMP Family of RNA-Binding Proteins

IMP2 belongs to the IMP family, which includes IMP1, IMP2, and IMP3 [4]. IMP1 and IMP2 share over 70% sequence identity; however, only 56% homology is shared among all three members [6]. IMP1 and IMP2 possess an equivalent number of RNA sequence motifs, including two RNA recognition motifs (RRM) and four hnRNP K homology (KH) domains [6]. Despite structural similarities, IMP2 binds to a distinct set of RNA targets and consensus binding motifs, as compared to IMP1 or IMP3 [6]. Unlike IMP1 and IMP3, which are typically expressed only during developmental stages, IMP2 persists into adulthood, displaying expression throughout the entire lifespan [6].

**Figure 2:**
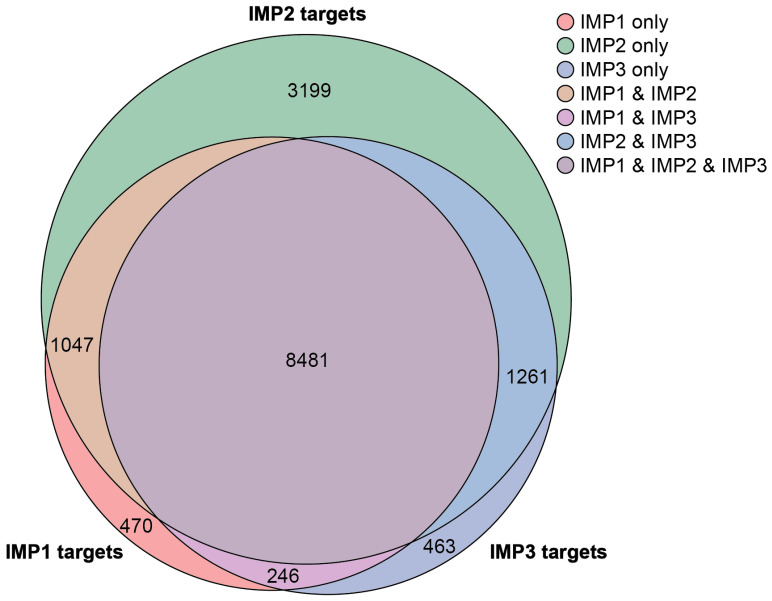
Venn diagram showing the number of overlapping and independent target RNAs regulated by IMP1, IMP2, and IMP3. CLIP-seq and other sequencing data from the POSTAR3 database [10]. Figure created in Python 3.11.0 using the matplotlib_venn library and Adobe Illustrator version 27.9.

Despite their similar structures, each IMP protein exhibits distinct functional roles, leading to differential regulation of target genes and physiological processes. Structurally, IMP1, IMP2, and IMP3 share high sequence homology, each containing two RNA recognition motifs and four KH homology domains. A comparative analysis of CLIP-seq data from the POSTAR3 database, incorporating datasets across multiple cell types, identified 15,167 unique genes collectively targeted by the IMPs, with 3199 genes—approximately 20%—being unique to IMP2 (Figure 2) [7]. IMP proteins can bind the mRNA of other IMP proteins, suggesting an additional layer of interaction between family members [7]. The specificity of target selection among IMP family members is influenced by variations in the KH3/4 domains [6]. Techniques such as systematic evolution of ligands by exponential enrichment (SELEX) and mutational functional validation have further elucidated their RNA-binding preferences. Evolutionary analysis suggests that IMP1 likely emerged first, playing an essential role in organismal survival, while IMP2 evolved to specialize in metabolic regulation. Notably, variations in RNA-binding sequences appear to have co-evolved alongside IMP proteins, supporting the idea that as IMP functions diversified, their RNA targets also adapted. IMP1, IMP2, and IMP3 proteins play crucial roles during embryogenesis, where they regulate metabolism and stem cell renewal [8]. While IMP1 and IMP3 are typically expressed at low levels in adult tissues, IMP2 is retained in several adult mouse organs [9].

The importance of IMP proteins is further highlighted in knockout (KO) mouse models, which exhibit both overlapping and distinct phenotypes, underscoring their context-dependent roles in development, metabolism, and disease. IMP1 KO mice display severe growth retardation, elevated perinatal mortality, and significant defects in intestinal development [11]. At the embryonic stage (E12.5), these mice exhibit marked downregulation of Igf2 translation, while the postnatal intestine shows reduced expression of extracellular matrix transcripts, including procollagen transcripts, indicative of impaired intestinal morphogenesis [11]. Brain development is also hindered in IMP1 KO mice. However, intestinal epithelial cell-specific deletion of Imp1 (Imp1ΔIEC) reveals a protective role in colonic repair, as these mice exhibit enhanced recovery from dextran sodium sulfate (DSS)-induced colonic injury. This recovery is accompanied by increased autophagy flux, Paneth cell granule changes, and upregulation of Atg5, suggesting that IMP1 is involved in posttranscriptional regulation of epithelial repair mechanisms [12].

In cancer models, IMP3 KO mice demonstrated that IMP3 had a critical role in colorectal cancer (CRC) progression. IMP3 directly bound the 3′-UTR of MEKK1 mRNA, stabilizing it and activating the MEK1/ERK signaling pathway, thereby promoting CRC malignancy. Notably, IMP3^−/−^ mice exhibit reduced MEKK1 expression and tumor burden following azoxymethane and dextran sodium sulfate treatment [13]. In contrast to the oncogenic role of IMP3, deletion of IMP1 in stromal cells in the same treatment model leads to increased tumor burden, suggesting an alternative tumor-suppressive function in certain tissue contexts [14].

Overall, the IMP family represents a group of multifunctional RNA-binding proteins with crucial roles in development, metabolism, and cancer progression. While all three IMPs play significant roles, IMP2 stands out due to its unique retention in adult tissues and its emerging implications in tumor biology as described below. Therefore, this review will delve deeper into the role of IMP2 in malignancies, exploring its molecular functions, regulatory mechanisms, and potential as a therapeutic target.

### 1.2. Mechanisms of Action

Recently, mass spectrometry and X-ray scattering identified that full-length IMP2 forms multiple oligomeric states; however, in the absence of RNA, it is primarily a dimer [15]. Specifically, it forms a head-to-tail dimer, where the tail of the first IMP2 molecule—containing the last 12 to 15 residues of the C-terminus, including the KH4 domain—binds to the head of the second IMP2 molecule, which includes the beginning of the N-terminus and RRM1. The KH3/4 domains have an anti-parallel pseudo-dimer orientation. The IMP2 sequence itself has many charged amino acids, especially at the termini, which allows for ionic interactions, such as binding between VKQQE and ELHGK [15]. In a purified in vitro system, the addition of RNA led to a larger conformational change while still maintaining IMP2 dimerization and was more stable and thermodynamically favorable compared to unbound IMP2 dimer. In this system, the RNA that IMP2 bound was likely unfolded; however, in full cellular systems, the folding conditions and RNA structural elements may vary [15].

The interaction between IMP2 and its target RNA transcripts depends on m6A modifications [16]. m6A (N6-methyladenosine) is a prevalent post-transcriptional modification found in mRNA and non-coding RNA in eukaryotic cells [17]. This modification is a dynamic and reversible process, regulated by the coordinated action of writers, erasers, and readers [18]. The primary writers of m6A are part of a multiprotein methyltransferase complex, which includes METTL3, METTL14, and WTAP. METTL3 serves as the catalytic subunit responsible for transferring the methyl group to the adenosine base, while METTL14 functions as an RNA-binding platform that enhances the activity and specificity of METTL3. WTAP ensures proper localization and assembly of the METTL3/METTL14 or the METTL16 writer complex within the nuclear speckles [19,20]. Additional components such as RBM15, KIAA1429, and ZC3H13 have been identified as part of the larger m6A writer complex [21,22,23]. The m6A modification can be removed by erasers, mainly FTO and ALKBH5 [24,25]. Reader proteins, including IGF2BP proteins and the YTH domain family (e.g., YTHDF1, YTHDF2, YTHDF3, and YTHDC1) proteins, recognize m6A-modified transcripts and modulate their stability and translation [2,26]. Phylogenetically conserved RRM domains on readers are responsible for IMP2 identifying m6A marks on RNA [17,27]. The impact of m6A is evident in both the nuclear and cytoplasmic compartments. In the nucleus, m6A is written on nascent transcripts and influences splicing and nuclear export, while in the cytoplasm, m6A modulates mRNA translation, stability, and localization [18,28].

Along the length of mRNAs, m6A modifications are not uniformly distributed. These modifications are most enriched in the 3′ untranslated region (UTR), with significant concentrations also found near the stop codon in the coding sequence (CDS) [29,30]. The overall density of m6A in the CDS is lower compared to the 3′ UTR, while the 5′ UTR generally shows the lowest frequency of these modifications. This distribution pattern is largely consistent across different cell types and tissues [30,31]. The distribution patterns of m6A in other RNA types are more varied and often specific to each RNA class [32].

This enrichment of m6A modifications in the 3′ UTR is particularly relevant to the function of IMP2 as an m6A reader. IMP2 preferentially binds to m6A-modified sites in the 3′ UTR, which promotes the formation of ribonucleoprotein (RNP) complexes, stabilizing the mRNA and preventing its degradation (Figure 1) [2]. Additionally, IMP2 interacts with m6A-modified sites in the CDS, where it can modulate mRNA stability and translation rates [2]. To a lesser extent, the protein binds to sites in the 5′ UTR, reflecting the distribution of m6A across mRNA regions.

IMP2 directly binds to the eIF4A protein, a member of the eIF4F translation initiation complex that acts as the helicase responsible for unwinding RNA secondary and tertiary structures to allow the ribosome to more efficiently translate mRNA into protein [33,34]. The circularization of mRNA during translation involves interactions between poly(A)-binding protein (PABP) and the cap-binding complex. This circularization is facilitated by the 3′ UTR and enhances translation efficiency by bringing the 5′ and 3′ ends of the mRNA into proximity, allowing for the regulation of translation through 3′ UTR binding sites [35]. While IMP2 primarily acts on the 3′ UTR, and eIF4A acts on the 5′ UTR, poly(A)-binding protein binds to both the 3′ poly-adenylated tail of messenger RNAs and the 5′ translation initiation complex, creating looped mRNAs that promote continuous translation. This suggests a mechanism by which IMP2 may regulate protein translation in an eIF4A-dependent manner.

Alternatively, when IMP2 binds to the 5′ UTR, as in the case of *IGF2* mRNA, it facilitates 5′ cap-independent translation through an internal ribosomal entry site (IRES) [36]. This mechanism is favored during cellular stress when cap-dependent translation is impaired. mTOR, via rapamycin-sensitive phosphorylation of IMP2 at Ser162 and Ser164, enhances binding of IMP2 to the *IGF2* L3 5′ UTR, promoting its IRES-mediated translation [36]. Experiments using dicistronic mRNA constructs—which contain one ORF whose translation is regulated by the canonical cap-dependent mechanism and a second ORF whose translation is regulated by IRES-mediated translation—showed that IMP2 enables 5′ cap-independent translation of *IGF2* L3 mRNA [36]. This process was dependent on mTOR signaling, which is also typical for IRES-mediated translation.

Additional mechanisms of action for IMP2 have been proposed (Figure 1). IMP2 may act to promote alternative splicing of its targets. For instance, overexpression of *IGF2BP2* cDNA caused a significant shift in the splice isoform ratio of IMP2 target *NFIC* [37]. Another possible mechanism is the binding of IMP2 to target genes to inhibit the attachment and subsequent action of other RNA-binding proteins or regulatory RNAs on the target sequences. It would be informative to investigate whether the knockdown of IMP2 restricts the binding of alternative RNA-binding proteins or regulatory RNAs at these sites, thereby influencing the expression of target mRNAs.

Finally, in a subset of RNAs, IMP2 has been reported to decrease mRNA stability. Specifically, IMP2 has been shown to promote the degradation of mRNAs, such as *progesterone receptor* (*PR*) and *Frizzled class receptor 8* (*FZD8*), by recruiting the CCR4-NOT deadenylase complex through interaction with its scaffold protein CNOT1 [38,39]. In adipocyte-derived stem cells from IMP2 conditional knockout mice, *FZD8* mRNA was highly elevated, indicating that IMP2 normally facilitates its degradation [39]. This process was found to be modulated by nutrient availability via mTOR-dependent phosphorylation of IMP2. Phosphomimetic mutants of IMP2 enhanced *FZD8* mRNA degradation, while phosphodeficient mutants resulted in its stabilization. Similarly, in triple-negative breast cancer (TNBC) cells, IMP2 and IMP3 were found to cooperate to destabilize *PR* mRNA by recruiting the CCR4-NOT complex, leading to reduced expression of *miR-200a* and promoting metastasis through epithelial–mesenchymal transition [38]. These findings suggest that nutrient availability–regulated phosphorylation may alter the mechanism of action of IMP2, enabling it to modulate cellular processes by promoting the degradation of specific target mRNAs through the CCR4-NOT complex.

### 1.3. Regulation of IMP2 Expression

IMP2 expression is impacted by diet and age. Mice subjected to fasting display decreased IMP2 protein in their livers and white adipose tissue [40]. Moreover, IMP2 expression declined as hematopoietic progenitor cells (HPCs) aged, and similarly, germline knockout of IMP2 in mice led to diminished growth and repopulation capacity in young HPCs, along with decreased markers of stemness—both phenotypes associated with HPC aging [41].

**Figure 3:**
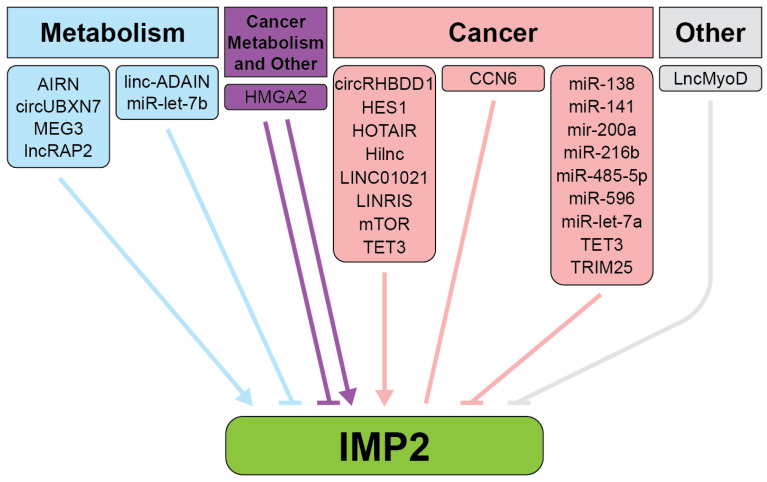
Upstream regulators of IMP2. Blue represents metabolism, pink represents cancer, and purple represents metabolism, cancer, and other indications. Figure created in Adobe Illustrator version 27.9.

DNA methylation and binding of transcription factors at the DNA promoter region upstream of the *IGF2BP2* gene can regulate its expression. In TNBC, TET3, a DNA demethylase, reduces methylation levels in the promoter region of *IGF2BP2*, facilitating its overexpression. This hypomethylation is a distinguishing feature of TNBC compared to other breast cancer subtypes [42]. Another regulator of IMP2 is the transcriptional regulator HMGA2 and its tumor-specific truncated form, HMGA2Tr (Figure 3, Table S1) [43]. HMGA2 is highly expressed during development, where it plays a critical role in regulating IMP2 expression, particularly through binding to AT-rich regions on the first intron of *IGF2BP2* in cooperation with NF-kB [43]. In adulthood, HMGA2 expression decreases but is re-expressed in diseases, including cancer and diabetes, where its effects are context-dependent. For example, during embryogenesis, HMGA2 upregulates IMP2 expression, as demonstrated by significantly reduced IMP2 levels in HMGA2-deficient mouse embryos, while IMP1 and IMP3 remain unaffected. However, in certain developmental contexts, the tumor-specific truncated form HMGA2Tr downregulates IMP2. In cancers such as embryonic rhabdomyosarcoma (ERMS), HMGA2 upregulates IMP2, stabilizing *NRAS* mRNA and sustaining oncogenic NRAS signaling. Additionally, in cancers with aberrant expression of the transcribed pseudogene *RPSAP52*, HMGA2 enhances the HMGA2-IMP2-RAS axis, promoting IMP2 binding to its mRNA targets and driving translation [43,44,45,46,47].

The ability of IMP2 to act upon its targets is influenced by long non-coding RNAs. For example, *lncRNA Airn* binds and stabilizes IMP2 for normal heart function. However, loss of this interaction leads to diabetic cardiac fibrosis [48]. Similarly, *Airn* protects against diabetic nephropathy (DN) by interacting with *IGF2BP2* mRNA to promote the translation of proteins like IGF2 and LAMB2, maintaining podocyte viability and glomerular barrier function [49]. When *Airn* is downregulated in DN, this interaction is lost, leading to reduced cell viability, increased apoptosis, and structural deficiencies. Another example is *lncRAP2*, a conserved cytoplasmic lncRNA enriched in adipose tissue [50]. *lncRAP2* directly binds to IMP2, forming a complex that stabilizes target mRNAs encoding key regulators of adipogenesis and energy metabolism, such as adiponectin. Downregulation of either *lncRAP2* or IMP2 disrupts this process, hindering adipocyte lipolysis and contributing to metabolic dysfunctions associated with obesity and diabetes.

In cancer, the long noncoding RNA, *LINC021*, binds to IMP2 to enhance the stability of m6A-modified transcripts such as *MSX1* and *JARID2* [51]. This interaction promotes CRC tumorigenesis by supporting cell proliferation, migration, and colony formation while reducing apoptosis. High levels of *LINC01021* in CRC tissues are associated with poor prognosis. The long noncoding RNA *HOTAIR* also impacts CRC progression by regulating IMP2 expression [52]. RNA interference-mediated silencing of *HOTAIR* decreases IMP2 levels, reducing proliferation, invasion, and migration of LoVo colon cancer cells while enhancing apoptosis. This regulation alters epithelial–mesenchymal transition markers, including decreased vimentin and increased E-cadherin, and suppresses tumor growth and microvessel density in vivo. Overexpression of IMP2 reverses these effects. *LINRIS* is another lncRNA associated with CRC [53]. *LINRIS* prevents K139 ubiquitination of IMP2, stabilizing the protein and preventing its degradation via the autophagosome-lysosome pathway. By maintaining IMP2 levels, *LINRIS* promotes *MYC*-mediated glycolysis and tumor progression in CRC. Inhibition of *LINRIS* reduces IMP2 levels, impairing CRC cell growth in vitro and tumor proliferation in vivo.

Conversely, *IGF2BP2* mRNA is negatively regulated by *lncMyoD*, which binds to IMP2 and prevents IMP2 from acting upon its targets to increase translation, allowing for exit out of the cell cycle and skeletal muscle differentiation [48,54]. Multiple microRNAs act as negative regulators of IMP2 across a variety of biological processes and diseases, including breast, pancreatic, and hepatocellular carcinoma, as well as keratinocyte migration, demonstrating the extensive regulatory network controlling its activity [55,56,57,58,59,60,61].

Dysregulation of IMP2 expression is associated with pathogenesis in various diseases, including metabolic disorders and cancer.

## 2. Metabolism

**Figure 4:**
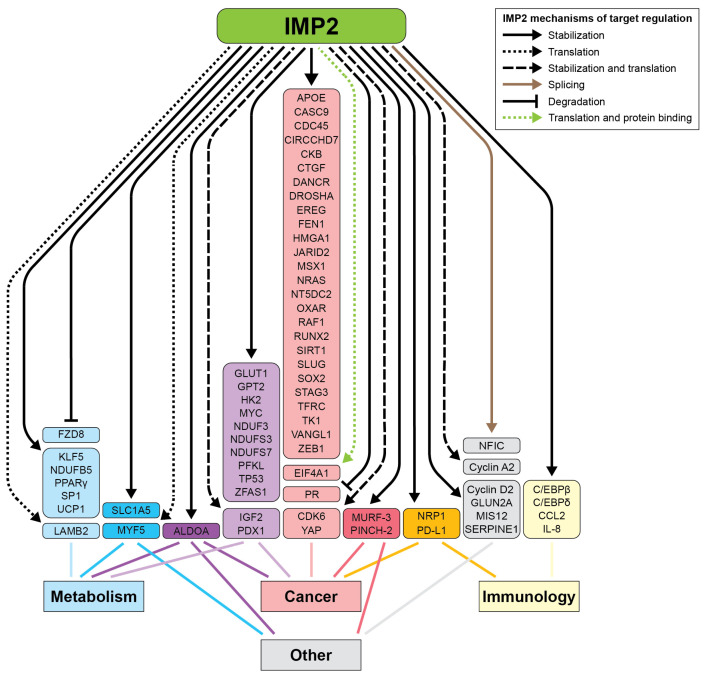
Validated targets of IMP2 and the disease in which the relationship was reported. Solid lines: IMP2 promotes RNA stability; dotted lines: IMP2 promotes translation; dashed lines: IMP2 promotes both RNA stability and translation; tan line: IMP2 regulates alternative splicing; inhibition symbol: IMP2 promotes RNA degradation; green dotted line: IMP2 promotes both translation of the messenger RNA and directly binds the protein product. Light blue: implicated in metabolism; dark blue: metabolism and other functions; dark purple boxes: metabolism, cancer, and other functions; light purple: metabolism and cancer; light pink: cancer; dark pink: cancer and other functions; orange: cancer and immunology; grey: other functions; yellow: implicated in immunology. Figure created in Adobe Illustrator version 27.9.

IMP2 plays a pivotal role in cellular metabolism, influencing key aspects of both normal physiology and disease states. It serves as a critical regulator of several metabolic pathways across different tissues (Figure 4, Table S2). Dysregulation of IMP2 is linked to several metabolic dysfunctions, including wound healing, type 2 diabetes (T2D), obesity, fatty liver disease, and cancer, highlighting its potential as a therapeutic target [13].

### 2.1. Adipose Tissue Metabolism, Obesity, and Related Disorders

IMP2 plays a complex role in adipose tissue metabolism and obesity. IMP2 promotes the commitment of mesenchymal stem cells (MSCs) to the adipogenic lineage [39]. Mice with PDGFRα promoter-regulated IMP2 deletion in MSCs (PIMP2-KO) showed resistance to diet-induced obesity. This effect is attributed to IMP2 regulation of glycolytic metabolism in adipocyte precursors, affecting their differentiation potential. Moreover, IMP2 is crucial for stabilizing the mRNAs of *IL-8* and *KLF5*—key genes involved in adipogenesis—upon *linc-ADAIN* knockdown in human adipocytes [62]. When *linc-ADAIN* is depleted, IMP2 shows an increased binding affinity for *IL-8* and *KLF5* mRNAs, enhancing their stability and preventing their decay. This results in elevated mRNA and protein levels of IL-8 and KLF5, which subsequently promotes adipocyte differentiation, lipid accumulation, and inflammation. Additionally, IMP2 acts as a downstream effector of *lncRNA MEG3* in regulating adipocyte inflammation and insulin [63]. IMP2 upregulation leads to increased expression of TLR4, which subsequently activates the IκKβ/NF-κB signaling pathway. This activation results in the promotion of inflammatory responses and the dysregulation of insulin sensitivity in adipocytes. Further, *Hilnc*, a long noncoding RNA regulated by the Hedgehog signaling pathway, interacts with IMP2 to regulate *PPARγ* mRNA stability, influencing lipid metabolism and hepatic steatosis [64]. *Hilnc* knockout or mutation in its promoter region confers resistance to diet-induced obesity and hepatic steatosis in mice.

Tissue-selective overexpression of IMP2 in murine livers led to steatosis and cirrhosis. Transgenic overexpression of IMP2 in mice using the liver-specific promoter LAP caused improved glucose tolerance, a buildup of liver fat, and cirrhosis [65,66,67]. While murine livers overexpressing IMP2 did not show changes in *IGF2* mRNA, they did display a loss of *PTEN*, an increase in p-AKT, and an increase in NFκ*B*, suggesting that other IMP2 targets may be responsible for these phenotypes. IMP2-overexpressing livers displayed de-differentiation markers, including increased CDH1, increased production of extracellular matrix components, and increased ductular reaction involving strings of liver progenitor cells [65].

When the *IGF2BP2* gene was knocked out specifically in pancreatic β-cells using a RIP2 promoter-driven Cre, β-cell proliferation and insulin secretion were decreased, leading to slower glucose processing [68]. On high-fat diets, β-cell IMP2 knockout mice had significantly faster liver fatty acid metabolism [68]. The IMP2 KO mice gained less weight and had less body fat than control mice, leading to longer overall lifespans [68].

### 2.2. Diabetes

IMP2 has been established as a significant player in the pathophysiology of type 2 diabetes (T2D). The association between IMP2 and T2D was first identified through genome-wide association studies (GWAS) in 2007, with three independent research groups demonstrating a strong link between genetic variants in the *IGF2BP2* gene and T2D risk [69,70,71]. These studies identified single-nucleotide polymorphisms (SNPs) in the second intron of the *IGF2BP2* gene, particularly rs4402960 and rs1470579, as being significantly associated with an increased risk of T2D across various populations. Individuals in a Han Chinese population carrying the TT genotype at rs4402960 had a higher risk of developing T2D compared to those with TG or GG genotypes, and CC carriers at rs1470579 were more susceptible to T2D than A allele carriers [72]. However, not all studies have replicated these associations. Research conducted in a French Caucasian population did not find a significant association between rs4402960 or rs1470579 and T2D risk [73]. More recent studies have continued to explore the role of IGF2BP2 in T2D. A meta-analysis published in 2024 indicated a notable association between the IGF2BP2 rs4402960 polymorphism and susceptibility to T2D under an over-dominant model. The study also suggested a link between the rs1470579 variant and T2D under allelic and recessive models [74]. It has not been determined whether SNPs in the IMP2 region are causative of T2D phenotypes or whether they share haplotype blocks with other causative elements.

IMP2 is integral to the development of T2D primarily through its effects on pancreatic β-cell function and insulin secretion. IMP2 promotes pancreatic β-cell proliferation and insulin secretion by enhancing PDX1 expression [68]. Mice with pancreatic β-cell-specific deletion of IMP2 (βIMP2KO) exhibited impaired insulin secretion and reduced compensatory β-cell proliferation when challenged with a high-fat diet (HFD) [68]. Mechanistically, IMP2 directly binds to *Pdx1* mRNA in an m6A-dependent manner, promoting its translation and stabilizing PDX1 protein through the IGF2-AKT-GSK3β-PDX1 signaling pathway [68]. This aligns with human studies showing that individuals with T2D-associated *IGF2BP2* variants have reduced insulin secretion [75].

Metabolic reprogramming, particularly the shift towards aerobic glycolysis, is recognized as one of the hallmarks of cancer. This phenomenon, also known as the Warburg effect, enables cancer cells to rapidly generate energy and biomass precursors even in the presence of oxygen, supporting their accelerated proliferation and survival in harsh tumor microenvironments [76]. Next, we will discuss the roles of IMP2 in driving several forms of cancer.

## 3. Cancer

**Figure 5:**
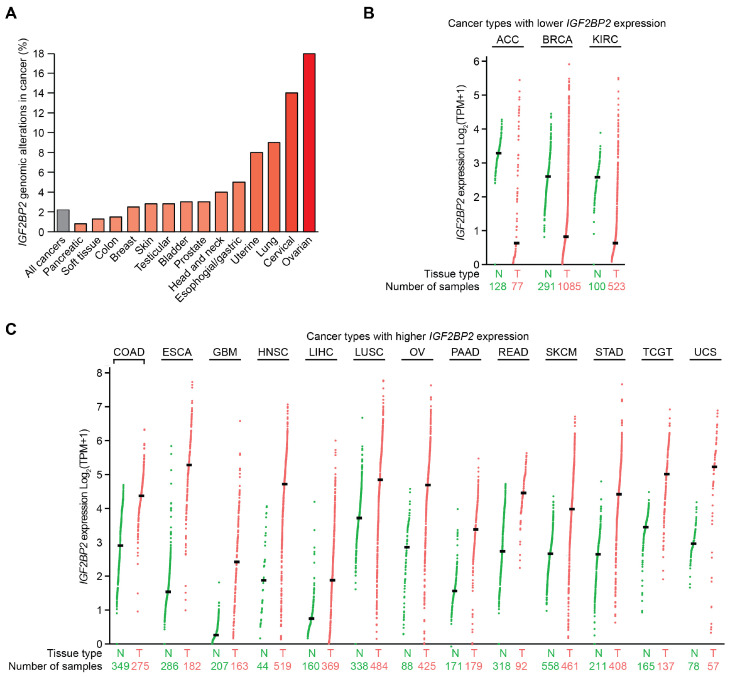
Genomic and transcriptional changes to *IGF2BP2* in cancer. (**A**) Rate of *IGF2BP2* genomic alterations in tumor samples. Data from the cBioPortal curated set of non-redundant studies [77,78,79]. (**B**) Cancers with decreased expression relative to normal tissue of *IGF2BP2* messenger RNA in log_2_(TPM+1) from tumor (T) relative to normal tissue (N) from the GEPIA2 database [81]. Log fold change between tumor and normal tissue: ACC (−2.66), BRCA (−1.78), and KIRC (−1.95). (**C**) Cancers with increased expression relative to normal tissue of *IGF2BP2* messenger RNA in log_2_(TPM+1) from tumor (T) relative to normal tissue (N) from the GEPIA2 database [81]. Log fold change between tumor and normal tissue: ESCA (3.74), HNSC (2.85), UCS (2.27), GBM (2.16), PAAD (1.94), OV (1.84), STAD (1.77), READ (1.73), TGCT (1.56), COAD (1.47), SKCM (1.32), LIHC (1.13), LUSC (1.12). See the Abbreviations section for definitions of acronyms. Figure adapted in Adobe Illustrator version 27.9.

IMP2 is an oncogene that modulates the expression of multiple proteins implicated in cancer progression. IMP2 is genomically altered in over 2% of all cancers, which is above the baseline of random alterations in the average passenger genes (Figure 5A) [77,78,79]. In a cBioPortal curated set of non-redundant samples, the *IGF2BP2* gene was altered in 18% of ovarian cancers, 14% of cervical cancers, 9% of lung cancers, 8% of uterine cancers, 5% of esophageal and gastric cancers, 4% of head and neck cancers, 3% of prostate cancers, 3% of bladder cancers, 2.8% of testicular cancers, 2.8% of skin cancers, 2.5% of breast cancers, 1.5% of colon cancers, 1.3% of soft tissue tumors, and 0.8% of pancreatic cancers (Figure 5A) [77,78,79]. In patient tumor samples, amplifications were most common, followed by point mutations, with very few genomic deletions. *IGF2BP2* mRNA and protein are frequently overexpressed across a wide variety of cancer types, as exemplified by proposals for IMP2 expression to be used as a predictive biomarker in prostate cancer as part of a panel of tumor-associated antigens [80]. These findings from primary patient tumor samples are consistent with IMP2 acting as a proto-oncogene activated by overexpression in many cancer settings (Table 1).

Differentially expressed oncogenes play a crucial role in cancer progression and patient outcomes. A pan-cancer analysis of *IGF2BP2* mRNA expression derived from GEPIA2 revealed significantly increased expression in 13 tumor types relative to normal tissues and decreased expression in three tumor types [81]. *IGF2BP2* mRNA expression was decreased in adrenocortical carcinoma (ACC), breast invasive carcinoma (BRCA), and kidney renal cell carcinoma (KIRC) compared to their respective normal tissue controls, with log fold changes between tumor and normal tissue measuring −2.66 (ACC), −1.78 (BRCA), and −1.95 (KIRC), respectively (Figure 5B). In contrast, increased *IGF2BP2* mRNA expression was observed for esophageal carcinoma (ESCA log fold change 3.74), head and neck squamous cell carcinoma (HNSC; 2.85), uterine carcinosarcoma (UCS; 2.27), glioblastoma multiforme (GBM; 2.16), pancreatic adenocarcinoma (PAAD; 1.94), ovarian serous cystadenocarcinoma (OV; 1.84), stomach adenocarcinoma (STAD; 1.77), rectum adenocarcinoma (READ; 1.73), testicular germ cell tumors (TGCT; 1.56), colorectal adenocarcinoma (COAD; 1.47), skin cutaneous melanoma (SKCM; 1.32), liver hepatocellular carcinoma (LIHC; 1.13), and lung squamous cell carcinoma (LUSC; 1.12) (Figure 5C). In most cancers analyzed, *IGF2BP2* mRNA was frequently overexpressed.

**Figure 6:**
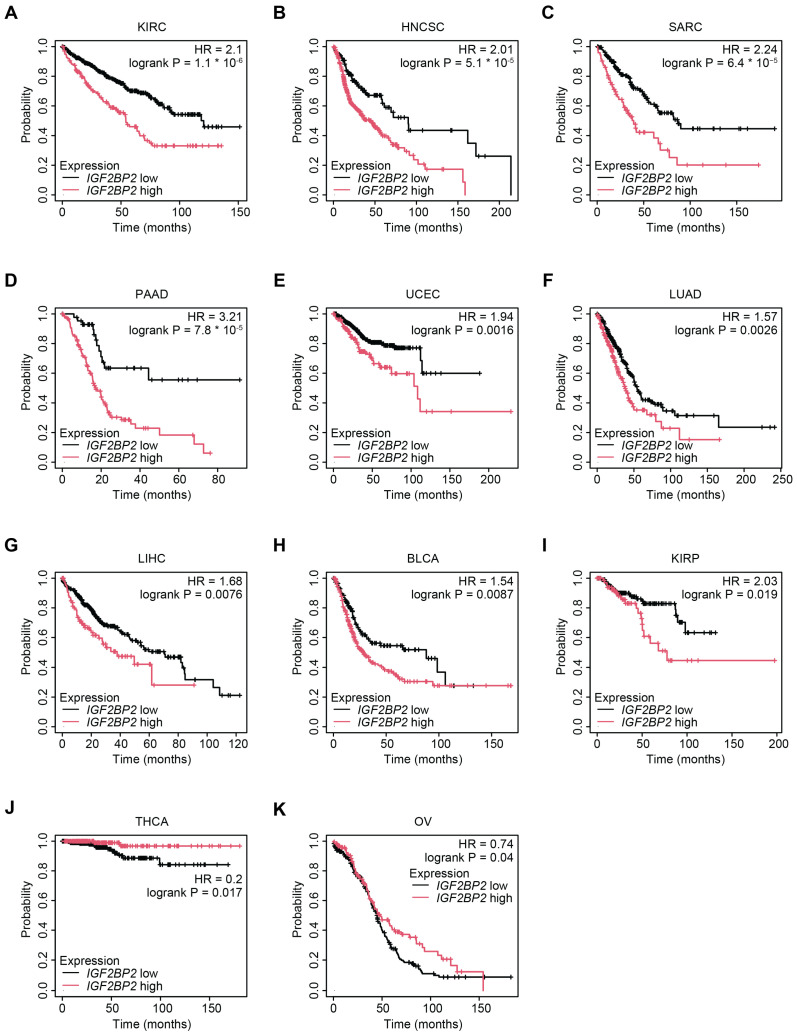
Kaplan–Meier analysis of cancers where higher *IGF2BP2* expression is significantly associated with decreased (**A**–**I**) or increased (**J**–**K**) overall survival; data was obtained from Kaplan–Meier Plotter [82,83]. Patients were stratified into high and low *IGF2BP2* expression groups based on median expression of *IGF2BP2*. Only cancers where *IGF2BP2* is significantly associated with survival are displayed: KIRC (**A**), HNCSC (**B**), SARC (**C**), PAAD (**D**), UCEC (**E**), LUAD (**F**), LIHC (**G**), BLCA (**H**), KIRP (**I**), THCA (**J**), and OV (**K**). Please see the Abbreviations section for definitions of acronyms. Figure adapted in Adobe Illustrator version 27.9.

The differential expression of IMP2 in cancers suggests its potential as a prognostic biomarker for patients. Survival analyses for *IGF2BP2* mRNA expression carried out using Kaplan–Meier Plotter found increased *IGF2BP2* expression was significantly associated with worse overall survival for multiple cancer types [82,83]. Specifically, in KIRC (HR = 2.1, log-rank < 0.0001), HNSC (HR = 2.01, log-rank < 0.0001), sarcoma (SARC) (HR = 2.24, log-rank < 0.0001), PAAD (HR = 3.21, log-rank < 0.0001), uterine corpus endometrial carcinoma (UCEC) (HR = 1.94, log-rank < 0.01), lung adenocarcinoma (LUAD) (HR = 1.57, log-rank < 0.01), LIHC (HR = 1.68, log-rank < 0.01), bladder urothelial carcinoma (BLCA) (HR = 1.54, log-rank < 0.01), and kidney renal papillary cell carcinoma (KIRP) (HR = 2.03, log-rank < 0.05) (Figure 6A–I). However, in select cancers, such as OV (HR = 0.74, log-rank < 0.05) and thyroid carcinoma (THCA) (HR = 0.2, log-rank < 0.05), higher *IGF2BP2* was correlated with increased survival (Figure 6J,K). Though overall in most cancers analyzed, high expression of *IGF2BP2* mRNA was associated with worse overall survival, thereby supporting the oncogenic role of IMP2 across a broad range of tumor types.

The functionally relevant targets of IMP2 have been characterized in several tumor subtypes, with some targets shared and others having been demonstrated in a limited number of model systems (Figure 4 and Table 1). For instance, across many cancer subtypes, IMP2 promotes cell growth and proliferation by binding and stabilizing the mRNA of HMGA1, a chromatin remodeling protein and established oncogene. HMGA1 suppresses IGFBP2, which normally binds and sequesters IGF2, allowing more IGF2 to reach and bind to its respective receptors, InsulinR-A and IGF1R [84].

Here, we will cover the key findings and effectors of IMP2 action in colorectal cancer, breast cancer, leukemia and lymphoma, hepatocellular carcinoma, lung adenocarcinoma, ovarian malignancies, endometrial cancer, oral cancer, renal cell carcinoma, thyroid cancer, and glioblastoma.

### 3.1. Colorectal Cancer

IMP2 is significantly overexpressed in colorectal cancer (CRC) cells and patient samples, highlighting its upregulation in the disease. Knockout of IMP2 in CRC cell lines demonstrated that heterozygous deletion led to proliferative deficiency [85]. There are several known targets of IMP2 that are important for the progression of colorectal cancer, including *MYC* and *RAF1* [86,87]. Autoantibody responses to IMP2 epitopes expressed in colon cancer were observed at a higher frequency compared to non-cancerous samples, underscoring the importance of IMP2 overexpression as a marker for malignancy [88]. For example, IMP2 binds near an m6A motif on the *Yes-associated protein* (*YAP*) mRNA [89]. This increases the expression of YAP protein. YAP is a positive regulator of HER2 expression, which acts to control enzymes critical for post-translational modifications and is often reactivated as a mechanism of resistance to inhibition of KRAS and other MAPK pathway proteins [90,91,92,93,94].

Colorectal cancer cells typically exhibit the Warburg effect, characterized by increased glucose uptake and glycolysis even in the presence of oxygen. IGF2 dysregulation in diabetes similarly alters glucose metabolism and enhances glucose influx, reflecting a shared feature between diabetes and CRC. IMP2 is a critical regulator of glycolysis in colorectal cancer, driving tumor progression by stabilizing m6A-modified mRNAs of key glycolytic enzymes, *hexokinase 2* (encoding HK2) and *SLC2A1* (encoding GLUT1) [16]. In CRC cell lines, IMP2 knockdown significantly reduces *HK2* and *GLUT1* mRNA levels and stability. This leads to decreased HK2 activity and lower lactate production, indicating reduced glycolytic flux. HES1 acts as an upstream regulator of *IGF2BP2* in CRC by directly binding to its promoter and increasing *IGF2BP2* mRNA levels. HES1 knockdown reduces GLUT1 levels, aerobic glycolysis, and CRC proliferation, while IMP2 is required for HES1-mediated stabilization of m6A-modified *GLUT1* mRNA, underscoring the importance of the HES1-IMP2 axis in CRC progression [95]. The interaction between IMP2 and its target transcripts depends on m6A modifications introduced by METTL3. In METTL3-knockout cells, IMP2 binding to *HK2* and *GLUT1* mRNAs is substantially impaired [16]. These results were further validated in vivo using xenograft mouse models, where manipulation of METTL3 expression affects tumor size and glucose uptake, highlighting the crucial role of the METTL3-IMP2 axis in CRC progression [16]. RNA-seq and MeRIP-seq identified *HK2* as a downstream target of IMP2, and MeRIP confirmed the interaction between m6A and *HK2* mRNA [96]. Both FTO and ALKBH5 m6A demethylases are significantly downregulated in CRC, particularly in patients who are obese, leading to increased m6A modification of *HK2* mRNA. This modification, mediated by IMP2, stabilizes *HK2* mRNA, promoting glycolysis and CRC proliferation.

Not only does IMP2 bind mRNAs that encode proteins, but it also can bind to long non-coding RNAs (lncRNAs), expanding the scope of its reach. Downstream of IMP2, the long non-coding RNA (lncRNA) *ZFAS1* is identified as a critical target stabilized by IMP2 [97]. IMP2 interacts with *ZFAS1* in an m6A-dependent manner, binding specifically to the m6A-modified adenosine at position +843 within the RGGAC/RRACH element of *ZFAS1*. Stabilized *ZFAS1* then interacts with OLA1, recognizing its OBG-type functional domain and facilitating the exposure of ATP-binding sites. This interaction boosts the activity of OLA, leading to increased ATP hydrolysis and glycolysis [97]. The IMP2-ZFAS1-OLA1 axis is instrumental in CRC progression by stabilizing *ZFAS1* and enhancing OLA1 activity, which in turn accelerates glycolytic metabolism and supports cancer cell proliferation and survival [97].

**Table 1:** Summary of the roles of IMP2 across cancer types (Section 3.1, Section 3.2, Section 3.3, Section 3.4, Section 3.5, Section 3.6, Section 3.7, Section 3.8, Section 3.9, Section 3.10 and Section 3.11), highlighting key findings, reported targets, and patient data. Patient data were derived from GEPIA2, Kaplan–Meier Plotter, and cBioPortal. A comprehensive list of reported targets is available in Table S2.

Cancer Type	Key Findings	Reported Targets	Patient Data
**Colorectal Cancer**	▪ IMP2 stabilized MYC, RAF1, and YAP mRNAs, driving tumor progression [86,87,89]. ▪ IMP2 promoted glycolysis, ATP hydrolysis, and tumor growth by stabilizing HK2 and GLUT1 [13,16,97].	GLUT1; HK2; JARID2; MSX1; MYC; RAF1; SOX2; STAG3; TFRC; YAP; ZFAS1	Overexpressed [81,85], genomic alterations 1.5% [77,78,79].
**Breast Cancer**	▪ SNP variants in *IGF2BP2* associated with breast cancer [98]. ▪ IMP2 regulates CDK6 expression, affecting the G1/S phase transition [42]. ▪ IMP2 stabilizes CTGF mRNA, promoting cell migration [99]. ▪ CCN6 regulates IMP2 in metaplastic breast carcinoma [100].	CDK6; CTGF; DROSHA; EIF4A1; MYC; PR	Underexpressed [81], genomic alterations 2.5% [77,78,79].
**Leukemia and Lymphoma**	▪ IMP2 stabilized lncRNA DANCR, enhancing PKM/HIF-1α signaling, increasing HK2 and GLUT1 expression [101]. ▪ In AML, IMP2 stabilized MYC, GPT2, and SLC1A5, promoting glutamine uptake and TCA cycle [101]. ▪ IMP2 KO in mice improves survival and delays leukemia progression [101,102]. ▪ IMP2 regulates proliferation through NT5DC2 in B-cell lymphoma [103].	DANCR; GPT2; MYC; SLC1A5; NT5DC2	Not available
**Hepatocellular Carcinoma**	▪ IMP2 drives liver fibrosis, enhancing the translation and stability of ALDOA [104]. ▪ IMP2 promotes liver cancer cell migration via Wnt/β-catenin activation [105]. ▪ IMP2 enhances CDC45 expression; depletion of CDC45 reduces tumor growth [101].	ALDOA; CDC45; FEN1; OXAR	Overexpressed [81], decreased survival [82,83].
**Lung Cancer**	▪ In NSCLC, CircDHTKD1 interacts with IMP2 to stabilize PFKL, promoting tumor growth and glycolysis [106].	VANGL1; PFKL; TK1	Genomic alterations 9% [77,78,79], overexpressed LUSC [82,83], decreased survival LUAD [81].
**Ovarian Cancer**	▪ IMP2 knockdown inhibits proliferation [107]. ▪ IMP2 is implicated with PCOS, promoting aberrant cell growth [37].	Not available	Genomic alterations 18% [77,78,79], overexpressed [81], increased survival [82,83].
**Endometrial Cancer**	▪ circCHD7 stabilizes PDGFRB via IMP2, activating JAK/STAT signaling—promoting endometrial cancer proliferation [28].	CIRCCHD7	Decreased survival [82,83].
**Oral Cancer**	▪ IMP2 stabilized EREG, promoting EMT [108]. ▪ IMP2 stabilized HK2, promoting tumor metabolism [109]. ▪ IMP2 associated with immune suppression [110].	EREG; HK2	IMP2 significantly upregulated in OSCC [109].
**Renal Cell Carcinoma**	▪ IMP2 suppressed metastasis by stabilizing CKB, reducing motility and invasion [111].	CKB	Underexpressed [81], decreased survival [82,83].
**Thyroid Cancer**	▪ IMP2 stabilizes APOE mRNA, activating the IL-6/JAK2/STAT3 pathway—promoting tumor growth and glycolysis [102].	ALDOA; RUNX2	Increased survival [82,83].
**Glioblastoma**	▪ IMP2-stabilized CASC9 enhances HK2 mRNA stability, promoting glycolysis [13].	CASC9; HK2; NDUFS3; NDUFS7; NDUF3	Overexpressed [81], decreased survival [13].

### 3.2. Breast Cancer

Single nucleotide polymorphism variants in the *IGF2BP2* gene have been associated with the incidence of breast cancer, including triple negative breast cancer (TNBC), with higher IMP2 protein expression consistent with an oncogenic role [98]. Through ELISA, IMP2 has been shown to trigger an autoimmune response in breast cancer, as evidenced by the presence of autoantibodies [99], suggesting the potential of screening for IMP2 as a biomarker for breast cancer. In TNBC, IMP2 plays a significant role in cell cycle progression. IMP2 affects the G1/S phase transition by binding to the m6A site of *CDK6* and regulating its expression. Unlike its best-characterized role in stabilizing mRNA targets, IMP2 regulates *CDK6* by recruiting the translation initiation factor eIF4A1 [42]. Additionally, the knockdown of IMP2 increased TNBC sensitivity to the CDK4/6 inhibitor abemaciclib, suggesting that an IMP2 inhibitor could be part of a combination regimen with a CDK6 inhibitor in the treatment of TNBC. Another target of IMP2 in breast cancer is *connective tissue growth factor* (*CTGF*). Immunoprecipitation and mRNA half-life assays demonstrated that IMP2 stabilizes CTGF mRNA, which contributes to promoting cell migration, a critical step in cancer metastasis [112]. In MDA-MB-468 cells, overexpression of IMP2 increased MYC mRNA and protein, whereas IMP2 knockout decreased both MYC mRNA and protein levels [112]. Moreover, functional assays, such as wound healing experiments, demonstrated that IMP2 overexpression enhances cell migration and reduces cell adhesion—hallmarks of invasive and migratory breast cancer.

In metaplastic breast carcinoma (MBC), CCN6 acts as an upstream regulator of *IGF2BP2* [100]. Loss of CCN6 in mammary-specific knockout mice leads to upregulation of IMP2 and increased tumor aggressiveness, while restoring CCN6 reduces IMP2 levels and tumor growth. In human models, CCN6 knockdown promotes invasion via IMP2, while IMP2 knockdown reverses this effect. Clinical samples confirm a correlation between low CCN6 and high IMP2 in spindle and squamous MBC, highlighting a CCN6/IMP2 axis with therapeutic implications and illustrating the diverse mechanisms through which IMP2 contributes to breast cancer progression.

### 3.3. Leukemia and Lymphoma

IMP2 supports glycolytic functions in FLT3-ITD-positive acute myeloid leukemia (AML), a common and aggressive form of AML characterized by an internal tandem duplication mutation in the *FLT3* gene [101]. IMP2 binds to m6A-modified sites on the lncRNA *DANCR*, preventing its degradation and thereby increasing its stability, which is significant as *DANCR* is upregulated in FLT3-ITD^+^ AML and is associated with poor prognosis [101]. *DANCR* functions as a competing endogenous RNA (ceRNA), sponging up *miR-4701-5p*, sequestering this microRNA, and preventing it from downregulating PKM (pyruvate kinase M), a critical enzyme in glycolysis. The resulting higher levels of PKM subsequently activated HIF-1α-dependent transcription of glycolytic enzymes such as *HK2* and *GLUT1*. This regulatory cascade is evidenced by the fact that *IGF2BP2* knockdown reduces HIF-1α reporter activity, while its overexpression increases it, leading to enhanced glycolysis.

Additionally, low IMP2 expression downregulates *HK2* and *GLUT1* mRNA levels in the B-cell leukemia cell line MV-4-11 cells, further confirming the role of IMP2 in promoting glycolysis through the PKM/HIF-1α pathway [101]. In B-cell lymphoma, IMP2 acts upon the cell cycle and regulates proliferation by binding to and stabilizing *NT5DC2*, which is involved in the metabolism of nucleotides by hydrolysis and is implicated in the progression of cancer [103].

In acute myeloid leukemia (AML), IMP2 stabilizes key mRNAs, including *MYC*, *GPT2*, and *SLC1A5*, that are essential for glutamine uptake and metabolism, fueling the TCA cycle and supporting AML cell proliferation and survival [113]. *IGF2BP2* is notably overexpressed in leukemia stem cells (LSCs), correlating with poor prognosis in patients with AML. This elevated expression is driven by upstream factors such as MLL fusion proteins, which are common in AML. RNA sequencing and m6A-sequencing analyses identify *MYC*, *GPT2*, and *SLC1A5* as direct targets of IMP2, showing that their stability and translation depend on m6A modifications. Functional experiments further reveal that knockdown of *IGF2BP2* in AML cells leads to reduced glutamine metabolism, impaired TCA cycle function, and decreased ATP production, while overexpression of *IGF2BP2* enhances these metabolic processes. Additionally, in vivo bone marrow transplantation assays in mice demonstrate that *IGF2BP2* knockout significantly delays AML progression, reduces leukemia cell engraftment, and prolongs survival.

### 3.4. Hepatocellular Carcinoma

Liver fibrosis is a wound healing response whose signaling is entwined with hepatocellular carcinoma [114]. IMP2 is highly expressed in liver fibrosis and activated hepatic stellate cells (HSCs), where it plays a pivotal role by modulating glycolytic metabolism [104]. Notably, inhibiting IMP2 protects against HSC activation and the progression of liver fibrogenesis involved in wound healing and growth. IMP2 enhances the stability and translation of Aldolase A (ALDOA), a key glycolytic enzyme crucial for HSC activation. Furthermore, the heightened glycolytic activity in these activated HSCs leads to substantial lactate production, which serves as a substrate for histone lactylation—a process essential for both initiating and maintaining the activated state of HSCs [104].

IMP2 is frequently overexpressed in hepatocellular cancer (HCC) and liver cancers. Knockout of IMP2 in hepatocellular carcinoma cell lines demonstrated that heterozygous deletion led to proliferative deficiency. CDC45, a protein important for DNA replication and cell division, is stabilized on the mRNA level in an m6A-dependent manner by IMP2 in HCC. Not only does IMP2 play a role in regulating cell growth and proliferation, but it is also involved in promoting cell migration, another hallmark of malignant cancers. While the direct mechanism is unknown, IMP2 overexpression activates the Wnt pathway, resulting in the relocalization of β-catenin from the cytoplasm to the nucleus, as well as the expression of Wnt3a, a Wnt ligand [105].

IMP2 promotes glycolysis in hepatocellular carcinoma (HCC) by stabilizing *CDC45* mRNA through m6A modification, which increases its expression [115]. High levels of CDC45 are associated with poor prognosis in patients with HCC, and knocking down *CDC45* inhibits malignant behaviors of HCC cells, including glycolysis. Specifically, knockdown of *CDC45* results in lower expression of glycolysis-related proteins such as HIF-1α, HK-2, GLUT1, and PKM2. Additionally, lactic acid and ATP levels are substantially lower in cells with reduced CDC45 expression, while glucose levels remain higher. Further, depletion of CDC45 impairs HCC cell growth, motility, invasiveness, and epithelial–mesenchymal transition, while simultaneously enhancing apoptosis. RIP assays confirmed that IMP2 directly interacts with *CDC45* mRNA in an m6A-dependent manner, enhancing its stability, as further supported by actinomycin D assays showing decreased mRNA stability upon IMP2 knockdown. In vivo xenograft models demonstrated that CDC45 depletion significantly reduces tumor growth, underscoring the critical role of the IMP2-CDC45 axis in driving HCC progression [115].

### 3.5. Lung Cancer

Genomic amplifications creating extra copies of *IGF2BP2* occur in 9% of lung cancers (Figure 5A) [77,78,79]. Homozygous *IGF2BP2* knockout in lung cells led to a complete halt of proliferation [85]. IMP2 promotes glycolysis and tumor growth in non-small cell lung cancer (NSCLC) by stabilizing the mRNA of the glycolytic enzyme PFKL. *CircDHTKD1*, a circular RNA, binds to IMP2, which in turn binds to *PFKL*, preventing its degradation [106]. *CircDHTKD1* in NSCLC cells led to enhanced IMP2-mediated stabilization of *PFKL*. This interaction is validated through RNA pull-down and RIP assays, showing that both *circDHTKD1* and *PFKL* bind to IMP2. Knockdown of IMP2 leads to increased degradation of *PFKL* mRNA, confirming its role in stabilizing *PFKL*. Additionally, Eukaryotic initiation factor 4A-III (EIF4A3) upregulates *circDHTKD1* in NSCLC cells, leading to enhanced IMP2-mediated stabilization of *PFKL*. As expected, *circDHTKD1* knockdown reduces *PFKL* mRNA and protein levels, while overexpression of *circDHTKD1* increases them, highlighting the importance of the IMP2-*circDHTKD1* interaction in promoting glycolysis in NSCLC cells [106]. In patients with NSCLC treated with immune checkpoint inhibitors, IMP2 expression is correlated with longer progression-free survival, suggesting that it might function as a prognostic biomarker [116].

### 3.6. Ovarian Cancer

Examination of immunohistochemical samples of various types of ovarian cancers, probing for CTGF expression of IMP1, IMP2, and IMP3, revealed that IMP2 was expressed in over 5% of all tumor types analyzed, with the highest levels observed in the micropapillary variant of serous borderline tumors and high-grade serous carcinoma (HGSCs), where expression ranged from 98 to 100% [117]. Protein expression profiling and quantitative protein analysis confirmed that IMP2 was statistically significantly expressed in HGSCs. Furthermore, survival assays showed that knockdown of IMP2 by siRNA decreased proliferation of HGSC ovarian lines. Flow cytometry demonstrated that the knockdown of IMP2 led to an increase in G0/G1 phase compared to the S phase, underscoring the importance of IMP2 in regulating cell proliferation in ovarian cancer [107].

IMP2 is implicated in polycystic ovarian syndrome (PCOS), a condition typified by infertility, inflammation, irregular menstrual cycles, multiple ovarian cysts, and an increased risk of progression to ovarian cancer [37]. In a PCOS model, overexpression of IMP2 in ovarian granulosa cells led to aberrant cell growth. IMP2 bound NFIC and promoted an alternative splice form associated with increased cell proliferation [37]. Furthermore, IMP2 overexpression in KGN cells, compared to controls, resulted in a higher number of novel alternative splicing events, highlighting its additional role as an RNA-binding protein. However, in the ovarian granulosa-like tumor cell line KGN, only a fraction of RNAs bound to IMP2 were differentially expressed and regulated by it, suggesting that the non-splicing functions of IMP2 may also play a significant role in these systems [37]. Targeting IMP2 could represent a new therapeutic strategy for treating patients with PCOS.

### 3.7. Endometrial Cancer

In endometrial cancer (EC), *circCHD7*, a circular RNA upregulated in tumor tissues, interacts with IMP2 to stabilize the mRNA of platelet-derived growth factor receptor beta (PDGFRB) [28]. The resulting increase in PDGFRB activates the JAK/STAT signaling pathway, driving cancer cell proliferation and survival. Circular RNAs, such as *circCHD7*, play important roles in RNA stability by often acting as sponges for microRNAs, preventing them from interacting with target mRNAs [28]. This emphasizes the broader role of circRNAs in cancer progression and highlights *circCHD7* as a potential target for therapeutic intervention in EC.

### 3.8. Oral Cancer

IMP2 is significantly upregulated in head and neck cancers, including oral squamous cell carcinoma (OSCC), where its elevated expression correlates with cancer progression and poorer survival rates [109]. IMP2 promotes epithelial–mesenchymal transition (EMT), a process critical for cancer cell invasion, by binding and stabilizing *EREG*, a regulator of EMT in OSCC and other malignancies [108]. This process is further supported by IMP2’s regulation of the FAK/SRC pathway, which controls phosphorylation levels of key proteins such as FAK, RAF, SRC, and MEK [108]. Additionally, high IMP2 expression is associated with immune suppression in oral cancer, reducing the number of CD8+ T cells in the tumor microenvironment [110].

In OSCC, IMP2 also drives tumor metabolism. Overexpression of IMP2 enhances glucose uptake, lactate production, and extracellular acidification rate—hallmarks of increased glycolysis [109]. By directly binding to the m6A-modified 3′-UTR of *HK2* mRNA, IMP2 stabilizes HK2 and elevates its protein levels, thereby accelerating aerobic glycolysis and tumor progression. Conversely, knockdown of IMP2 reverses these metabolic changes, impairing glycolysis and tumor growth. In vivo xenograft models further confirm that IMP2 knockdown suppresses tumor growth, underscoring its multifaceted role in driving OSCC progression [109].

### 3.9. Renal Cell Carcinoma

In clear cell renal cell carcinoma (ccRCC), IMP2 exhibits a unique role compared to its function in most cancers [111]. Unlike its frequent upregulation in other malignancies, IMP2 expression is significantly downregulated in ccRCC. Functional assays in vitro and in vivo demonstrate that IMP2 suppresses cell migration and invasion in ccRCC by binding to and stabilizing the mRNA of creatine kinase B (CKB), thereby enhancing its expression. CKB acts as a key inhibitor of metastatic behavior by reducing cell motility and invasive capacity. Conversely, knockdown of IMP2 reduces CKB levels, which correlates with increased metastatic potential in xenograft models [111]. These findings underscore the tissue-specific versatility of IMP2, revealing its unexpected role as a metastasis suppressor in ccRCC and its contribution to mitigating cancer progression in this context.

### 3.10. Thyroid Cancer

Luciferase reporter assays and RNA stability tests demonstrated that IMP2 stabilizes *Apolipoprotein E* (*APOE*) mRNA via m6A modification, increasing its expression [102]. This in turn activates the IL-6/JAK2/STAT3 signaling pathway, promoting glycolysis and tumor progression in papillary thyroid cancer (PTC). FTO, an m6A demethylase, is significantly downregulated in PTC tissues, and FTO knockdown resulted in increased expression of m6A-modified *APOE* mRNA. Comprehensive RNA-seq and MeRIP-seq have identified m6A-modified sites on *APOE* mRNA. Further validation through luciferase reporter assays and RNA stability tests confirmed that IMP2 binds to the m6A-modified *APOE* mRNA, increasing its stability and expression. These findings highlight the critical role of the FTO/APOE/IMP2 axis in regulating glycolysis in PTC, with IMP2 acting as a key stabilizer of *APOE* mRNA to enhance glycolysis and promote tumor growth [102].

### 3.11. Glioblastoma

The upregulation of IMP2 in glioblastoma multiforme (GBM) is associated with poor prognosis. In GBM, IMP2 recognizes the m6A modification site on the long non-coding RNA *CASC9*, enhancing its stability [13]. The interaction between IMP2 and *CASC9* was demonstrated through RNA stability assays and RIP qPCR. The IMP2/CASC9 complex increases the ability of IMP2 to bind and stabilize *HK2* mRNA, which in turn promotes glycolysis in GBM [13]. Correlation analysis indicated a positive relationship between IMP2 and HK2 expression in samples from patients with GBM [13]. Taken together, the diverse roles of IMP2 in promoting tumor progression—through stabilizing key mRNAs, regulating glycolysis, and influencing the tumor microenvironment—underscore its significance as a molecular driver of cancer across different tissues and tumor types.

## 4. Immunology

### 4.1. Inflammation

IMP2 promotes inflammatory responses in several autoimmune conditions, including autoimmune neuroinflammation and autoimmune glomerulonephritis (AGN), working in conjunction with cytokines like IL-17 and TNFα [118]. In experimental autoimmune encephalomyelitis (EAE), a model of multiple sclerosis, IMP2 stabilized *CCL2* mRNA, enhancing its expression and leading to the recruitment of myeloid cells to the central nervous system (CNS), exacerbating inflammation. IL-17 amplifies CCL2 expression, enhancing monocyte recruitment and promoting Th17 cell polarization, which collectively contribute to CNS inflammation. *IGF2BP2* −/− knockout mice exhibited reduced CCL2 production, impaired monocyte recruitment, and Th17 cell polarization, resulting in resistance to EAE. Similarly, in AGN, IMP2 stabilized mRNAs encoding the transcription factors C/EBPβ and C/EBPδ, driving the expression of inflammatory genes such as Lipocalin-2 (LCN2) [119]. TNFα further contributes to this process by inducing the expression of C/EBPβ and C/EBPδ, thereby promoting the inflammatory response. Knockout mice lacking IMP2 were resistant to AGN, exhibiting lower renal expression of C/EBPs and LCN2, which resulted in reduced inflammation and less kidney damage.

### 4.2. Macrophage Polarization

Beyond driving autoimmune inflammation, IMP2 plays a central, context-dependent role in macrophage polarization [120]. Expressed at high levels in both M1-like and M2-like phenotypes, IMP2 mediates inflammatory or anti-inflammatory responses depending on the signaling pathways activated. In the presence of IL-4, IMP2 was found to promote M2-like macrophage activation through the STAT6-HMGA2-IMP2 axis by stabilizing *TSC1* and *PPARγ* mRNAs. PPARγ is a key regulator of fatty acid metabolism, essential for the oxidative phosphorylation that drives M2-like polarization. TSC1 acts as a negative regulator of mTORC1, preventing its hyperactivation and thereby facilitating M2-like polarization while avoiding an unwanted shift to the M1-like phenotype. Additionally, IMP2 influenced M1-like macrophage polarization by stabilizing the TSC1/2 complex, which regulates the MEK/ERK signaling pathway, critical for M1-like activation. Therefore, the genetic deletion of *IGF2BP2* skewed macrophage polarization toward the M1-like phenotype by allowing unchecked mTORC1 activity and overactivation of the MEK/ERK pathway, disrupting the delicate balance required for proper immune responses. *IGF2BP2* knockout models exhibited reduced M2-like activation, impairing the resolution of allergic inflammation, while simultaneously increasing M1-like activation, which exacerbates inflammatory conditions such as colitis [120].

In the specific context of diabetic kidney disease (DKD), IMP2 has been identified as a pivotal regulator that stabilizes *SP1* mRNA, promoting macrophage infiltration and polarization toward the pro-inflammatory M1-like phenotype, which exacerbates renal inflammation and fibrosis [121]. The circular RNA *circUBXN7*, significantly upregulated in DKD, was shown to bind directly to IMP2, enhancing its ability to stabilize *SP1* mRNA and increase SP1 expression. The molecular interaction between *circUBXN7* and IMP2 was demonstrated through RNA pull-down and RNA immunoprecipitation assays, while the association between IMP2 and *SP1* mRNA was demonstrated through RIP assays. Notably, SP1 also transcriptionally upregulates *circUBXN7*, establishing a positive feedback loop that amplifies SP1 expression. In vivo experiments using a mouse model of DKD further supported these findings, revealing that manipulating *circUBXN7* expression affected macrophage behavior and contributed to disease progression [121]. Collectively, these results suggest that IMP2 plays a central role in regulating macrophage polarization in DKD by modulating SP1 expression, thereby driving inflammatory and fibrotic responses.

### 4.3. Immuno-Oncology

IMP2 plays diverse roles in shaping the immune landscape of various cancers, influencing tumor progression and immune evasion through multiple mechanisms, as demonstrated across different cancer models. In colorectal malignancies, IMP2 is a key regulator of tumor-associated macrophages (TAMs) by influencing the cargo of extracellular vesicles (EVs) released by cancer cells [122]. In vitro experiments, where EVs were isolated from human colorectal cancer cells (HCT116) and used to treat primary human monocyte-derived macrophages (HMDMs), revealed that IMP2 shapes the miRNA and protein composition of these EVs. When these EVs were taken up by macrophages, they drove polarization towards a TAM-like phenotype. Notably, EVs from IMP2-expressing cells were enriched in *miR-181a-5p*, a microRNA that downregulates DUSP6, a negative regulator of the MAPK signaling pathway. This downregulation facilitated the pro-tumorigenic activation of macrophages, leading to enhanced matrix degradation, upregulation of tumor-promoting genes, and the secretion of factors that support cancer cell migration. A comparison between EVs from IMP2-expressing cells and those from IMP2 knockout cells showed that EVs from knockout cells were less effective in driving these pro-tumorigenic changes. Additionally, in vivo experiments using a zebrafish embryo model confirmed that EVs from IMP2-expressing cells significantly altered macrophage behavior, reducing the expression of pro-inflammatory cytokines like TNF and IL-6.

Lung cancer, specifically NSCLC, represents another example of IMP2’s involvement in tumor progression and immune regulation [123]. *CircNDUFB2*, a circular RNA found to be downregulated in NSCLC, was shown to interact with this entire IMP family, facilitating their ubiquitination and degradation via the E3 ligase TRIM25. This degradation not only suppressed the oncogenic activities of these proteins but also initiated a strong immune response. The decrease in IMP levels activated the RIG-I-MAVS signaling pathway, leading to the production of type I interferons and pro-inflammatory cytokines like IFNβ. These immune signals recruited CD8+ T cells and dendritic cells into the tumor microenvironment, strengthening antitumor immunity. The interaction between *circNDUFB2* and IMPs was validated through RNA immunoprecipitation and RNA pull-down assays, while in vivo xenograft studies demonstrated that *circNDUFB2* overexpression reduced tumor growth and increased immune cell infiltration, highlighting the role of IMP2 in immune activation in NSCLC.

Gastric tumors exploit the ability of IMP2 to stabilize *PD-L1* mRNA to facilitate immune escape, a process mediated through its interaction with the circular RNA *circRHBDD1* [124]. PD-L1 is an immunosuppressive cell surface protein expressed on many tumors. Overexpressed in gastric cancer, *circRHBDD1* was found to bind to *IGF2BP2* and shield it from ubiquitination and degradation by interfering with its interaction with the E3 ligase TRIM25. The preservation of IMP2 levels enhanced the stability of *PD-L1* mRNA, leading to elevated PD-L1 expression. This upregulation suppressed CD8+ T cell infiltration and activity, allowing the tumor to evade immune surveillance. RNA immunoprecipitation and proteasome inhibition assays demonstrated this mechanism, while in vivo models using C57BL/6 mice showed that silencing *circRHBDD1* reduced tumor growth by enhancing CD8+ T cell responses, underscoring the critical involvement of IMP2 in the immune evasion strategies of gastric cancer.

In melanoma, IMP2, as part of the IMP family, plays a crucial role in regulating immune responses by binding to and stabilizing mRNAs coding for interferon-stimulated genes (ISGs) and major histocompatibility complex (MHC) class I molecules [125]. Knockout experiments using CRISPR/Cas9 in mouse melanoma models showed that the loss of *IGF2BP2* led to increased expression of MHC class I molecules, increasing cells’ ability to promote intracellular IFN-γ expression in syngeneic T-lymphocytes.

Suppression of immune responses associated with elevated IMP2 levels has been reported in oral squamous cell carcinoma (OSCC), particularly affecting CD8+ T cell infiltration within the tumor microenvironment [110]. Bioinformatic analyses indicated that high IMP2 expression correlates with lower immune, stromal, and microenvironment scores. This reduced immune presence is likely due to the role of IMP2 in regulating immune-related pathways and altering the tumor microenvironment to be less favorable for immune cell activity, possibly through the modulation of extracellular matrix components or cytokine profiles. Gene set enrichment analysis (GSEA) linked high IMP2 expression with the activation of pathways that promote cancer cell proliferation and immune suppression. Additionally, a negative correlation between IMP2 and CD8A expression suggests that IMP2 hinders the infiltration of CD8+ T cells, which are crucial for effective antitumor immunity.

These findings reveal the dual roles of IMP2 in promoting immune evasion in some cancers while facilitating immune activation in others, underscoring its complexity in immuno-oncology. Understanding the context-specific mechanisms of IMP2′s immune modulation could inform novel therapeutic strategies aimed at restoring immune surveillance or enhancing immune activation in the tumor microenvironment.

## 5. Conclusions

IMP2 simultaneously regulates multiple oncogenes and immunosuppressive factors, suggesting that IMP2 inhibitors could develop into anti-cancer therapeutics. Screening of compound libraries led to the identification of IMP2 inhibitors, which bound IMP2 by STD/NMR [126]. The anti-proliferative effects of these IMP2 inhibitor compounds on colorectal cancer and lung cancer cell lines were only partially rescued by biallelic knockout of *IGF2BP2*, suggesting that these compounds may have additional off-targets [85,126].

The IMP2 inhibitor CWI1-2 was identified by virtual screening for compounds predicted to bind IMP2 [113]. Hits were screened using the drug affinity responsive target stability (DARTS) assay for their ability to protect IMP2 in leukemic cell lysates, the cellular thermal shift assay for their ability to bind IMP2, and each compound’s capacity to inhibit leukemic cell growth [113]. CWI1-2 decreased oxygen consumption rates and extended survival in murine models of acute myeloid leukemia and mixed-lineage leukemia [113].

Overall, IMP2 plays key roles in normal development, diseases, and as a therapeutic target. It acts to increase target mRNAs through stabilization, translation, and/or promoting alternative splicing. IMP2 is a mechanistic player in diabetes, as inactivating polymorphisms in IMP2 can lead to decreased pancreatic insulin secretion [68,75]. *IGF2BP2* is itself a proto-oncogene, genomically amplified or overexpressed in a variety of malignant tissues (Figure 5 and Table 1). IMP2 causes upregulation of multiple pro-oncogenic targets that increase proliferation, contribute to the Warburg effect, and drive tumor cells toward immune escape [16,110,125]. The promise of pharmacologically inhibiting IMP2 opens new doors to studying and modulating this central regulatory RNA-binding protein, toward improving patient outcomes.

## Supplementary Materials

The supporting information can be downloaded at https://www.mdpi.com/article/10.3390/ijms26062415/s1. References [4,13,16,28,36,37,38,39,40,42,44,47,48,49,50,51,53,54,55,56,57,58,59,60,61,62,63,64,68,84,87,89,95,96,97,100,101,102,103,104,106,108,111,112,113,115,118,119,121,123,124,127,128,129,130,131,132,133,134,135,136,137,138,139,140,141,142,143,144,145,146,147,148,149,150,151,152,153] are cited in Supplementary Materials.

## Abbreviations

3′ UTR	3′ Untranslated Region
5′ UTR	5′ Untranslated Region
ACC	Adrenocortical Carcinoma
AGN	Autoimmune Glomerulonephritis
AIRN	Antisense of IGF2R Non-Protein Coding RNA
ALKBH5	AlkB Homolog 5 (m6A demethylase)
ALDOA	Aldolase A, Fructose-Bisphosphate
AML	Acute Myeloid Leukemia
APOE	Apolipoprotein E
BC	Breast Cancer
BCa	Bladder Cancer
BCSC	Breast Cancer Stem Cells
βIMP2KO	Beta-Cell-Specific IMP2 Knockout
BLCA	Bladder Urothelial Carcinoma
BRCA	Breast Invasive Carcinoma
C/EBPβ	CCAAT Enhancer Binding Protein Beta
C/EBPδ	CCAAT Enhancer Binding Protein Delta
CASC9	Cancer Susceptibility Candidate 9 (lncRNA)
CC	Cervical Cancer
ccRCC	Clear Cell Renal Cell Carcinoma
CCL2	C-C Motif Chemokine Ligand 2
CCR4-NOT	CCR4-NOT Transcription Complex
CCN6	Cellular Communication Network Factor 6 (WISP3)
CDC45	Cell Division Cycle 45
CDH1	Cadherin 1 (E-Cadherin)
CDK6	Cyclin-Dependent Kinase 6
CINC	Chemotherapy-Induced Neuropathic Pain
CIRCCHD7	Circular RNA CHD7 (Chromodomain Helicase DNA-Binding Protein 7)
CircDHTKD1	Circular RNA DHTKD1 (Dehydrogenase E1 And Transketolase Domain Containing 1)
CircNDUFB2	Circular RNA NDUFB2 (NADH Dehydrogenase (Ubiquinone) Oxidoreductase Subunit B2)
CircRHBDD1	Circular RNA RHBDD1 (Rhomboid Domain Containing 1)
CIRCUBXN7	Circular RNA UBXN7 (UBX Domain Containing Protein 7)
CKB	Creatine Kinase, Brain-Type
COAD	Colon Adenocarcinoma
CRC	Colorectal Cancer
CTGF	Connective Tissue Growth Factor
Cyclin A2	Regulator of Cell Cycle Progression, Encoded by the CCNA2 Gene
Cyclin D2	Regulator of Cell Cycle Progression, Encoded by the CCND2 Gene
DANCR	Differentiation Antagonizing Non-Protein Coding RNA (lncRNA)
DARTS	Drug Affinity Responsive Target Stability
DKD	Diabetic Kidney Disease
DLBCL	Diffuse Large B-Cell Lymphoma
DROSHA	Drosha Ribonuclease III
DUSP6	Dual Specificity Phosphatase 6
EAE	Experimental Autoimmune Encephalomyelitis (Model for Multiple Sclerosis)
EC	Endometrial Cancer
EIF4A1	Eukaryotic Translation Initiation Factor 4A1
EIF4A3	Eukaryotic Translation Initiation Factor 4A-III
EIF4F	Eukaryotic Initiation Factor 4F Complex (contains EIF4A)
EMT	Epithelial–Mesenchymal Transition
EREG	Epiregulin
ERMS	Embryonal Rhabdomyosarcoma
ESCA	Esophageal Carcinoma
EVs	Extracellular Vesicles
FEN1	Flap Endonuclease 1
FTO	Fat Mass and Obesity-Associated Protein (m6A Demethylase)
FZD8	Frizzled Class Receptor 8
GBM	Glioblastoma Multiforme
GC	Gastric Cancer
GluN2A	Glutamate Ionotropic Receptor NMDA Type Subunit 2A
GLUT1	Glucose Transporter 1
GPT2	Glutamic Pyruvate Transaminase 2
GSEA	Gene Set Enrichment Analysis
HCC	Hepatocellular Carcinoma
HCT116	Human Colorectal Cancer Cell Line
HES1	Hairy and Enhancer of Split 1
Hilnc	Hypoxia-Induced Long Noncoding RNA
HK2	Hexokinase 2
HMGA1	High Mobility Group AT-Hook 1
HMGA2	High Mobility Group AT-Hook 2
HMDMs	Human Monocyte-Derived Macrophages
hMSC	Human Mesenchymal Stem Cell
HNSCC or HNSC	Head and Neck Squamous Cell Carcinoma
HPCs	Hematopoietic Progenitor Cells
HSCs	Hepatic Stellate Cells
IFNβ	Interferon Beta
IGF1R	Insulin-Like Growth Factor 1 Receptor
IGF2	Insulin-Like Growth Factor 2
IMP2	Insulin-Like Growth Factor 2 mRNA-Binding Protein 2
IGFBP2	Insulin-Like Growth Factor-Binding Protein 2
IL-6	Interleukin 6
IL-8	Interleukin 8
InsulinR-A	Insulin Receptor A
ISGs	Interferon-Stimulated Genes
JAK2	Janus Kinase 2
JARID2	Jumonji, AT-Rich Interactive Domain 2
KGN	Human Ovarian Granulosa-Like Tumor Cell Line
KH	hnRNP K Homology (Domain)
KIRC	Kidney Renal Cell Carcinoma
KIRP	Kidney Renal Papillary Cell Carcinoma
KLF5	Kruppel-Like Factor 5
KOC	K Homology Domain Containing Protein Overexpressed in Cancer (Alternative Name for IGF2BP2)
KRAS	Kirsten Rat Sarcoma Viral Oncogene Homolog, Proto-Oncogene, GTPase
LAMB2	Laminin Subunit Beta 2
LCN2	Lipocalin-2
LIHC	Liver Hepatocellular Carcinoma
Linc-ADAIN	Long Intergenic Non-Protein Coding RNA Adipose Anti-Inflammatory
LINC01021	Long Intergenic Non-Protein Coding RNA 1021
LINRIS	Long Intergenic Non-Coding RNA Regulator of IGF2BP2 Stability
LncMyoD	Long noncoding RNA Myogenic Differentiation 1
LncRAP2	Long Non-Coding RNA Regulated in Adipogenesis 2
LncRNA HOTAIR	Long Non-Coding RNA HOX Transcript Antisense Intergenic RNA
LncRNA MEG3	Long Non-Coding RNA Maternally Expressed Gene 3
LSCC	Laryngeal Squamous Cell Carcinoma
LUAD	Lung Adenocarcinoma
LUSC	Lung Squamous Cell Carcinoma
m6A	N^6^-Methyladenosine modification on RNA
MAPK	Mitogen-Activated Protein Kinase
MAVS	Mitochondrial Antiviral-Signaling Protein
MBC	Metastatic Breast Carcinoma
MeRIP-seq	Methylated RNA Immunoprecipitation Sequencing
METTL14	Methyltransferase Like 14 (m6A Methyltransferase Complex Component)
METTL3	Methyltransferase Like 3 (m6A Methyltransferase Complex Component)
MHC	Major Histocompatibility Complex
miR-138	MicroRNA 138
miR-141	MicroRNA 141
miR-200a	MicroRNA 200a
miR-216b	MicroRNA 216b
miR-4701-5p	MicroRNA 4701-5p
miR-485-5p	MicroRNA 485-5p
miR-596	MicroRNA 596
miR-let-7a	MicroRNA let-7a
miR-let-7b	MicroRNA let-7b
MIS12	Missegregation 12 (Kinetochore Complex Component)
MSX1	Msh Homeobox 1
mTOR	Mechanistic Target of Rapamycin
mTORC1	Mechanistic Target of Rapamycin Complex 1
MURF-3	Muscle RING-Finger Protein-3
MV-4-11	Human B-Cell Leukemia Cell Line
MYC	Myelocytomatosis Proto-Oncogene
MYF5	Myogenic Factor 5
NDUF3	NADH Dehydrogenase (Ubiquinone) 1α Complex Assembly Factor 3
NDUFB5	NADH Dehydrogenase (Ubiquinone) Oxidoreductase Subunit B5
NDUFS3	NADH Dehydrogenase (Ubiquinone) Iron–Sulfur Protein 3
NDUFS7	NADH Dehydrogenase (Ubiquinone) Iron–Sulfur Protein 7
NF-κB	Nuclear Factor Kappa-Light-Chain-Enhancer of Activated B-Cells
NFIC	Nuclear Factor I C
NRAS	Neuroblastoma RAS Viral (V-Ras) Oncogene Homolog
NRP1	Neuropilin 1
NSCLC	Non-Small Cell Lung Cancer
NT5DC2	5′-Nucleotidase Domain Containing 2
OLA1	Obg-Like ATPase 1
OSCC	Oral Squamous Cell Carcinoma
OV	Ovarian Serous Cystadenocarcinoma
OXAR	Oxaliplatin Resistance–Related lncRNA in NASH-HCC
P53	Tumor Protein p53
PAAD	Pancreatic Adenocarcinoma
PABP	Poly(A) Binding Protein
PD-L1	Programmed Death-Ligand 1
PDAC	Pancreatic Ductal Adenocarcinoma
PDGFRB	Platelet-Derived Growth Factor Receptor Beta
PCOS	Polycystic Ovary Syndrome
PFKL	Phosphofructokinase, Liver Type
PI3K	Phosphoinositide 3-Kinase
PIMP2-KO	IMP2 Knockout in Mesenchymal Stem Cells
PINCH-2	Particularly Interesting New Cysteine-Histidine Rich Protein 2
PKM	Pyruvate Kinase M
PPARγ	Peroxisome Proliferator-Activated Receptor Gamma
PR	Progesterone Receptor
PTC	Papillary Thyroid Carcinoma
PTEN	Phosphatase and Tensin Homolog
RAF1	Rapidly Accelerated Fibrosarcoma-1
RBM15	RNA-Binding Motif Protein 15
RBPs	RNA-Binding Proteins
READ	Rectum Adenocarcinoma
RIG-I	Retinoic Acid-Inducible Gene I
RIP-qPCR	RNA Immunoprecipitation Quantitative Polymerase Chain Reaction
RMS	Rhabdomyosarcoma
RNP	Ribonucleoprotein
RR-PTC	Radioactive Resistant Papillary Thyroid Carcinoma
RRM	RNA Recognition Motif
RUNX2	Runt-Related Transcription Factor 2
SARC	Sarcoma
SERPINE1	Serpin Family E Member 1
SIRT1	Sirtuin 1
SKCM	Skin Cutaneous Melanoma
SLC1A5	Solute Carrier Family 1 Member 5
SLC2A1	Solute Carrier Family 2 Member 1
SLUG/SNAI2	Snail Family Transcriptional Repressor 2
SNPs	Single-Nucleotide Polymorphisms
SOX2	SRY-Box Transcription Factor 2
SP1	Specificity Protein 1
STAD	Stomach Adenocarcinoma
STAG3	Stromal Antigen 3
STAT3	Signal Transducer and Activator of Transcription 3
STD/NMR	Saturation Transfer Difference/Nuclear Magnetic Resonance
T2D	Type 2 Diabetes
TAM	Tumor-Associated Macrophage
TCA	Tricarboxylic Acid
TGCT	Testicular Germ Cell Tumors
TET3	Ten-Eleven Translocation Methylcytosine Dioxygenase 3
TFRC	Transferrin Receptor (CD71)
THCA	Thyroid Carcinoma
TK1	Thymidine Kinase 1
TNBC	Triple-Negative Breast Cancer
TNF	Tumor Necrosis Factor
TRIM25	Tripartite Motif Containing 25
TSC1	TSC Complex Subunit 1
UCEC	Uterine Corpus Endometrial Carcinoma
UCP1	Uncoupling Protein 1
UCS	Uterine Carcinosarcoma
VANGL1	Van Gogh-Like 1 (VANGL Planar Cell Polarity Protein 1)
VICKZ	Vg1 RBP/Vera; IMP-2; CRD-BP; KOC; ZBP-1 (Alternative Name for IGF2BP2)
WTAP	Wilms Tumor 1-Associating Protein (Part of m6A Writer Complex)
YAP	Yes-Associated Protein
YTHDC1	YTH Domain-Containing Protein 1
YTHDF1/YTHDF2/YTHDF3	YTH N6-Methyladenosine RNA-Binding Protein Family
ZEB1	Zinc Finger E-Box Binding Homeobox 1
ZFAS1	ZNFX1 Antisense RNA 1
